# Oncological and functional outcomes and complications of robotic intracorporeal Studer orthotopic neobladder: A single-center retrospective study

**DOI:** 10.14440/bladder.2024.0025

**Published:** 2025-01-09

**Authors:** Qiang Cheng, Yin Lu, Bin Jiang, Qing Ai, Fan Gao, Xupeng Zhao, Jinlu Tang, Yi Feng, Wenfeng Gao, Hongzhao Li

**Affiliations:** 1 Department of Urology, Chinese People’s Liberation Army General Hospital, Beijing 100039, China; 2 Chinese People’s Liberation Army Medical School, Beijing 100853, China; 3 Senior Department of Urology, The Third Medical Center of People’s Liberation Army General Hospital, Beijing 100039, China; 4 School of Medicine, Nankai University, Tianjin 300071, China

**Keywords:** Intracorporeal Studer orthotopic neobladder, Robotic surgeries, Oncologic outcomes, Functional outcomes, Complications

## Abstract

**Background:**

Robotic intracorporeal Studer orthotopic neobladder (RISON) is a complex procedure for bladder reconstruction.

**Objective:**

This study aimed to retrospectively examine the oncological/functional outcomes, and complications of RISON at a single center.

**Methods:**

Baseline data and perioperative results of patients who received RISON between March 2018 and December 2022 were analyzed. Sixty-two cases (60 males, 2 females), with a mean age of 56.79 ± 9.12 years, were included in the study. Follow-up data regarding RISON’s therapeutic effects, including tumor outcomes, neobladder capacity, continence, and complications, were collected.

**Results:**

All patients underwent the procedure without conversion to open surgery or changes in urinary diversion. The mean operative time lasted 379.2 ± 88.8 min, with a median blood loss of 200 mL (range: 100–300 mL). Indwelling time of the Ryles tube was 3.78 ± 2.23 days, and post-operative hospital stay was 10 days (range: 8–12 days). Pathological examination showed 87.1% (54/62) of cases had T2N0M0 tumors. A mean of 17.42 ± 8.03 lymph nodes were dissected, with three cases developing lymph node metastasis. Short-term complications (within 30 days) occurred in 51.9% of patients, while long-term complications (after 30 days) were found in 51.9% of patients. The mean neobladder volume measured 344.31 ± 147.00 mL. Daytime continence was achieved in 88.2% of patients, and night-time continence was attained in 39.2%. The average night-time urinary frequency was 2.78 ± 1.55 times, with 1.9 urine pads used on average. Follow-up duration ranged from 27 to 73 months, with a median time of 52.5 months. Five patients died of tumor metastasis, spreading to bone, liver, lung, brain, or lymph nodes. The 36- and 60-month cumulative recurrence-free survival rates were 96.3% and 87.4%, respectively. The 36- and 60-month cumulative overall survival rates were 96.3% and 90.4%, respectively.

**Conclusion:**

Clinically, RISON is a safe and feasible procedure with excellent oncological and functional outcomes, showing promise for widespread application.

## 1. Introduction

Bladder cancer represents the 10^th^ most common cancer across the globe, with approximately 573,000 new cases and 213,000 deaths in 2020.1 While the ileal conduit remains the most commonly used form of urinary diversion globally, there has been a notable increase in the adoption of orthotopic neobladder in recent years.2 Radical cystectomy is the standard treatment for muscle-invasive bladder cancer (MIBC) and high-risk recurrent non-muscle-invasive bladder cancer (NMIBC).^3^ Following a radical cystectomy, a urinary diversion is required, with common types including ileal conduit, orthotopic neobladder, and cutaneous ureterostomy.^4^,^5^ With advancements in robotic surgery technology and the growing emphasis on rehabilitation, intracorporeal urinary diversion has been gaining widespread application. Data from the International Robotic Cystectomy Consortium indicate that the utilization rate of intracorporeal urinary diversion increasing from 9% in 2005 to 97% in 2015, with the adoption rate of orthotopic neobladder rising from 7% to 17%.^6^ Randomized controlled trials have demonstrated that, compared to traditional open surgery, total laparoscopic urinary diversion results in comparable oncological outcomes but reduces the transfusion rate and offers certain quality-of-life benefits.^7^,^8^ In this study, we retrospectively reviewed the data from a single-center, prospective study of robotic intracorporeal Studer orthotopic neobladder (RISON). The efficacy of the procedure was evaluated with respect to oncological and functional outcomes, as well as complications. Oncological outcomes included post-operative pathology and recurrence rates, and functional outcomes were assessed based on neobladder capacity and daytime continence. Complications were evaluated and graded using the Clavien-Dindo classification system.

## 2. Materials and methods

### 2.1. Study population

RISON was performed on 62 patients by the same surgeon (Dr. HZ Li) from March 2018 to December 2022 at the General Hospital of the People’s Liberation Army (PLA), Beijing, China. A retrospective analysis was conducted using a prospectively maintained database of these patients. The study was approved by the Ethics Committee of the Chinese PLA General Hospital Third Medical Centre (approval number: 2018-P2-081-04). All patients provided written informed consent, and all relevant details were discussed with and consented to by the patients. Patients with missing data were excluded from the study. A total of 52 patients were effectively followed up.

### 2.2. Indications and perioperative care

All surgical procedures were performed in accordance with the guidelines for patients with non-metastatic MIBC, unresponsive NMIBC, and recurrence following bladder-sparing therapy.3 Nerve-sparing radical cystectomy was performed in patients who were sexually active and desired to conserve sexual function. The specific steps of the nerve-sparing technique are detailed in our previous study.9 Perioperative management followed the principles of enhanced recovery, including pre-operative counseling, early post-operative mobilization, and chewing gum on the first post-operative day.^10^,^11^ All relevant information was recorded in detail, including age, body mass index (BMI), whether patients received neoadjuvant chemotherapy, duration of indwelling Ryles tube, operative time, blood loss, post-operative hospital stay, tumor outcomes, neobladder capacity, incontinence, and occurrence of complications. Patients with locally advanced disease or positive surgical margins were given adjuvant chemotherapy.

### 2.3. Follow-up

Follow-up was conducted in accordance with the National Comprehensive Cancer Network/European Association of Urology (EAU) guidelines. Patients were recommended to undergo a computed tomography scan every 3–4 months during the 1^st^ year, and then every 6 months until the 3^rd^ year.^3^ We followed the EAU’s recommendations on the appropriate use of social media and maintained contact with each patient for rehabilitation and follow-up through outpatient visits, telephone, and WeChat.^12^ Daytime continence was defined as the ability to stay dry during the day without the need for urinary pads, while night-time continence was measured by the number of pads used. Night-time urination was defined as the number of times a person urinated between 22:00 and 06:00. All post-operative complications (early [≤30 days] and late [>30 days]) were graded using the Clavien–Dindo classification system, and the details of the complications were clearly recorded.

### 2.4. Statistical analysis

Continuous data are presented as median and interquartile range, while categorical data are expressed as frequencies and proportions. The Kaplan–Meier method was used to depict recurrence-free survival (RFS) and overall survival (OS). Data analysis was performed using IBM SPSS Statistics 26.0 (United States).

## 3. Results

**Figure 1:**
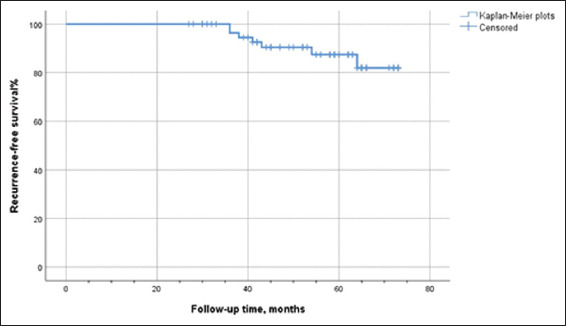
Kaplan–Meier plots of recurrence-free survival

**Figure 2:**
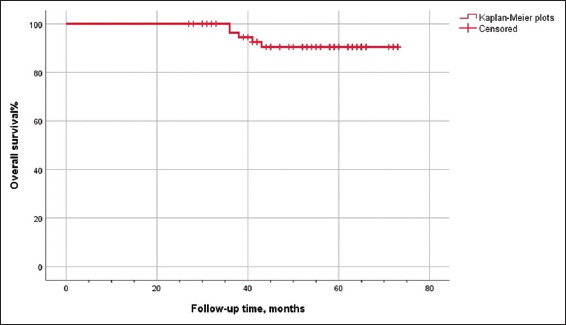
Kaplan–Meier plots of overall survival

All patients underwent RISON without conversion to open surgery or changes in urinary diversion. The cohort included 60 males and two females, with a mean age of 56.79 ± 9.12 years and a BMI of 25.31 ± 3.03 kg/m^2^. Neoadjuvant platinum-based chemotherapy was administered in 11 out of the 62 patients. The mean operative time lasted for 379.2 ± 88.8 min, with a median blood loss of 200 mL (range: 100–300 mL). The indwelling time for the Ryles tube was 3.78 ± 2.23 days, and the post-operative hospitalization duration was 10 days (range: 8–12 days). Post-operative pathological results revealed that 54 (87.1%) cases were staged T2N0M0. Of the 62 cases, three had a positive surgical margin, and one was diagnosed with incidental prostate cancer. A mean of 17.42 ± 8.03 lymph nodes were dissected, and three cases had confirmed lymph node metastasis (Table 1). Short-term (within 30 days) complications occurred in 51.9% (27/52) of patients, including Clavien Grade I in eight cases, Grade II in 15 cases, and Grade III in four cases. Long-term (beyond 30 days) complications also occurred in 51.9% (27/52) patients, with Clavien Grade I in 16 cases, Grade II in nine cases, and Grade III in two cases (Table 2). One patient underwent ileal conduit surgery 13 months postoperatively due to recurrent urinary tract and abdominal infections, hydronephrosis, and ascites. Another female patient developed incontinence 3 months after surgery and required self-catheterization at home. Excluding the patient who received ileal conduit diversion and the one requiring self-catheterization, the average bladder capacity of the remaining patients was 344.3 ± 47.00 mL. Daytime continence afflicted 88.2% (45/51) of patients, while 39.2% (20/51) maintained night-time continence. The average night-time urinary frequency was 2.78 ± 1.55 times, with patients using an average of 1.9 urine pads. Forty-five patients had night-time urinary intervals of <180 min (Table 3). The follow-up period ranged from 27 to 73 months, with a median of 52.5 months. Recurrence took place in two patients at 19 and 21 months post-surgery, at the ureter-neobladder junction and in the pelvic lymph nodes, respectively. Seven patients developed metastasis postoperatively. Five patients (four males and one female) died from tumor metastasis at 36, 36, 38, 41, and 43 months after surgery. Metastatic sites included the bone, liver, lung, brain, and lymph nodes. The 36- and 60-month cumulative RFS rates were 96.3% and 87.4%, respectively. The 36- and 60-month cumulative OS rates were 96.3% and 90.4%, respectively (Figures 1 and 2).

**Table 1:** Baseline and perioperative data

Characteristics	Value
Total, n (%)	62 (100)
Male	60 (96.8)
Female	2 (3.2)
Age, yr, mean±SD	56.79±9.12
BMI, kg/m^2^, mean±SD	25.31±3.03
Neoadjuvant chemotherapy, n (%)	
Presence	11 (17.8)
Absence	51 (82.2)
Pre-operative continence	Normal
Operation time, mins, mean±SD	379.2±88.8
Indwelling Ryles tube, days, mean±SD	3.78±2.23
Post-operative hospital stay, days, median (IQR)	10 (8–12)
pT stage, n (%)	
T0, Tis	3 (4.8)
T1	15 (24.2)
T2	36 (58.1)
T3	4 (6.5)
T4	4 (6.5)
Positive surgical margin, n (%)	3 (4.8)
Incidental prostate cancer, n (%)	1 (1.6)
pN stage, n (%)	
0	0
1	3 (4.8)
2	0
Number of lymph nodes, n, Mean±SD	17.42±8.03

Abbreviations: BMI: Body mass index; IQR: Interquartile range; mins: Minutes; pN: Pathological nodal status; pT: Pathological tumor extent; SD: Standard deviation; yr: Years.

**Table 2:** Complication outcomes

Complications	Number	Grade	Treatment
Early (≤30 days), n (%)	27 (51.9)	-	-
Infectious complications	15 (28.8)	II	Intravenous antibiotics
Gastrointestinal complications	5 (9.6)	I	Gastrointestinal decompression
Ascites	4 (7.7)	III	Abdominocentesis or surgery
Other complications	3 (5.8)	I	Observation
Late (>30 days), n (%)	27 (51.9)	-	-
Infectious complications	9 (17.3)	II	Oral or intravenous antibiotics
Gastrointestinal complications	4 (7.7)	I	Gastrointestinal decompression
Hernia	3 (5.8)	I	Observation
Hernia	1 (1.9)	III	Surgery
Hydronephrosis	8 (15.4)	I	Observation
Ascites	1 (1.9)	III	Surgery
Dysuria	1 (3.0)	I	Catheterization

Note: Continence is defined as the absence of the need for pads.

**Table 3:** Continence outcomes

Characteristics	Value
Total, n (%)	51 (82.3)
Bladder volume, mL, mean±SD	344.31±147.00
Day continence, n (%)	45 (88.2)
Night continence, n (%)	20 (39.2)
Night urine, n, mean±SD	2.78±1.55
Urine pads, n	1.9
Night-time urinary intervals<180 min, n (%)	45 (88.2)

Abbreviation: SD: Standard deviation.

## 4. Discussion

Bladder cancer is typically classified into NMIBC and MIBC. The optimal treatment for NMIBC involves complete resection of all visible lesions within the bladder, followed by intravesical instillation or early radical cystectomy based on risk stratification.^13^ For MIBC, radical cystectomy is widely regarded as the standard treatment, as endorsed by guidelines from the EAU, the American Urological Association, the American Society of Clinical Oncology, and the American Society for Radiation Oncology.^14^ After radical cystectomy, a urinary diversion is necessary, and its choice must consider both complications and tumor characteristics.^15^ Pre-operative diagnosis of infiltrative urethral cancer is considered a contraindication for neobladder reconstruction following bladder resection.^16^ Relative contraindications for an orthotopic neobladder include severe incontinence related to the urethral sphincter, complex urethral strictures, and high-dose pre-operative radiation therapy.^8^ Our center has also conducted research on and clinically used robotic bladder cancer resection and orthotopic neobladder surgery.

Due to the complexity and high technical demands, RISON was initially restricted to large, high-volume medical institutions. However, with the advancement and widespread introduction of the technology, intracorporeal neobladder diversion has been shown to yield satisfactory outcomes, in line with results from contemporary open surgical series.17 Furthermore, intracorporeal urinary diversion offers comparable complication rates, superior perioperative outcomes, and similar oncological outcomes compared to extracorporeal urinary diversion.18-21

Various techniques for intracorporeal neobladder reconstruction have been described, including the Karolinska-modified Studer neobladder,^22^ University of Sothern California-modified Studer neobladder,^23^ pyramid pouch,^24^ Y-pouch,^25^ and Vescica Ileale Padovana.26 The Studer orthotopic bladder is highly operable and offers advantages in terms of orthotopic bladder capacity and urinary control.^27^,^28^ Based on our experience and a review of previous cases, we have summarized several key technical considerations: (i) if the patient’s BMI is <24 kg/m^2^, we recommend positioning all trocars 1–2 cm to the cephalic side; (ii) strengthening the posterior urethral wall and Denonvilliers’ fascia with barbed sutures can lower the tension at the urethra-neobladder neck anastomosis and enhance the structural support around urethra, improving continence postoperatively; (iii) early anastomosis of the urethra and neobladder neck facilitates precision surgery under optimal visualization, maximizes urethral length, and provides additional fixation points at the distal end, thereby reducing bowel mobility and making intestinal management easier. In addition, this approach allows for more effective adjustment of the neobladder shape to morphologically fit the pelvic cavity; (iv) selecting the appropriate staple height and length is essential to ensure perpendicularity to the long axis of the intestine, thereby ensuring adequate mesentery attachment at the end of the intestine and preserving the blood supply to the bowel; (v) when suturing the neobladder, stitching the serosal and muscular layers together helps shape the bladder in a varus configuration, effectively preventing urine leakage. Proper positioning of the suture line improves the bladder shape, provides appropriate traction, and simplifies suturing, thereby reducing operative time; (vi) a no-tension, mucosa-to-mucosa anastomosis using a no-touch technique ensures optimal ureteral viability; (vii) since the procedure is entirely intracorporeal, the exposure time of the intestine is minimized, resulting in a faster functional recovery and allowing for valuable time for adjuvant chemotherapy, when needed.

The main challenges associated with this procedure include: (i) the surgical technique is complicated, requiring a relatively protracted operation time, particularly in the early stages. Surgeons must possess extensive experience in robotic surgery to perform the procedure effectively; (ii) the learning curve is steep, with a higher incidence of intraoperative complications observed during the early phase of the learning process; (iii) the procedure necessitates use of specialized surgical instruments, which can pose added financial burden on patients; (iv) post-operative care involves effective communication with patients and thorough rehabilitation guidance and follow-up. In addition, pre-operative imaging cannot accurately stage bladder tumors: The diagnostic accuracy for tumor outward growth ranges from 55 to 92%, and the sensitivity for detecting lymph node metastasis based solely on size is 48–87%.^29^-^31^ F-18 fluorodeoxyglucose positron emission tomography/computed tomography has been used to detect lymph node metastasis in bladder cancer, with a sensitivity of 0.57 (95% confidence interval: 0.49–0.64) and specificity of 0.92 (95% confidence interval: 0.87–0.95).^32^ Due to China’s healthcare insurance policies, out-of-pocket expenses for the same procedure may vary among patients, making it essential to discuss the surgical costs categorically. Given the small sample size of this study, the economic impact of RISON was not analyzed. Although robotic-assisted radical cystectomy (RARC) did not demonstrate a financial advantage over its traditional counterparts, it has been found to be associated with reduced overall perioperative blood transfusion rates compared to open surgery, with no significant differences in perioperative and post-operative complications or 3-year oncological outcomes between them.^33^ Consequently, the use of RARC plus total intracorporeal urinary diversion may present an unnecessary economic burden on patients and the benefits and risks should be carefully weighed. Therefore, patients for this procedure should be meticulously selected.

With surgeons gaining experience, intracorporeal neobladder diversion is incrementally being adopted and has shown potential benefits, including reduced fluid and blood loss, less pain, smaller surgical incisions, expedited recovery of bowel function, and fewer anastomotic strictures.34 In our study, the early complication rate (51.9%) was higher than 30–31.4% reported in the literature, and the late complication rate (51.9%) was also greater than those in previous studies.^17^,^24^ This finding might be attributed to the early cases being part of the learning curve,^35^ where patients treated early tend to experience worse perioperative and functional outcomes.^36^ However, after standardization of the procedure, significant improvements were noted in complication rates, hospital stays, and recovery of daytime continence. Wei *et al*.^37^ compared an enhanced recovery after surgery (ERAS) group with non-ERAS groups and found that ERAS implementation was associated with fewer complications (29.4% vs. 64.5%, *P* = 0.018), shorter time to first flatus (2 vs. 3 days, *P* = 0.016), and earlier initiation of liquid diet (2 vs. 4 days, *P* < 0.001) in intracorporeal urinary diversion. Furthermore, the nerve-sparing technique has proven to be advantageous in terms of both continence and erectile function in orthotopic neobladder procedures.^17^,^24^,^38^,^39^ Our study preliminarily confirmed these findings, though further larger-sample studies and longer time follow-ups are warranted to provide more accurate evidence.

Kretschmer *et al*.^40^ reported that, among 188 patients who underwent radical cystectomy and ileal orthotopic neobladder, good pre-operative Eastern Cooperative Oncology Group performance status (odds ratio 2.987, *P* = 0.010), retained sensation for bladder filling (odds ratio 6.462, *P* = 0.003), and the absence of pre-operative coronary heart disease (odds ratio 0.036, *P* = 0.002) were independent predictors of daytime continence.^40^ However, there is a lack of standardized assessment methods for this outcome, as well as an absence of objective outcome measurements through questionnaires or assessments of urinary dynamics. Clifford *et al*.^41^ developed a validated questionnaire to evaluate continence following radical cystectomy and orthotopic neobladder, finding that continence improved significantly by 6 months and subsequently plateaued, with 92% of patients achieving daytime continence by 12–18 months. Satkunasivam *et al*.^42^ compared urodynamics, tumor outcomes, and health-related quality of life (HRQOL), concluding that patients with intracorporeal neobladder had excellent urodynamic results and comparable bladder cancer-specific HRQOL scores to those with extracorporeal neobladder. More recent evidence suggests that functional outcomes following RISON are acceptable, but larger, prospective randomized trials are needed to confirm these findings.^43^

Our study is subject to several limitations. The sample size was relatively small, and there might be some loss of data. In addition, the study data were from a single medical center, which might limit the generalizability of the results. Further research on nerve-sparing techniques in RISON could provide more compelling evidence of its efficacy. Moreover, the number of female patients in our study was very small.

## 5. Conclusion

RISON is an increasingly viable option for urinary diversion, offering advantages such as smaller incisions, reduced pain, fewer complications, and faster post-operative recovery compared to traditional open surgery. This technique has the potential to significantly improve the quality of life for patients following urinary diversion. The continued adoption of RISON is expected to gain momentum and further enhance patients’ clinical outcomes and overall well-being.

## Data Availability

The data supporting the findings of this study are available from the corresponding author upon reasonable request.
